# The Kinase Mirk/dyrk1B: A Possible Therapeutic Target in Pancreatic Cancer 

**DOI:** 10.3390/cancers2031492

**Published:** 2010-07-14

**Authors:** Eileen Friedman

**Affiliations:** Upstate Medical University, State University of New York, Syracuse, New York, NY 13210, USA; E-Mail: friedmae@upstate.edu; Tel.:+1-315-464-7148

**Keywords:** Mirk, Dyrk, reactive oxygen species (ROS), K-ras

## Abstract

Pancreatic ductal adenocarcinomas are strongly resistant to chemotherapeutic drugs and radiation, underscoring the need for new therapeutic targets, particularly ones which target the numerous out of cycle cancer cells. Analysis of resected tumors for nuclear Ki67 antigen has shown that about 70% of pancreatic cancer cells are out of cycle, some post-mitotic. Other out of cycle cells are in a quiescent, reversible G0 state, resistant to drugs which target dividing cells, with some able to repopulate a tumor. The serine/threonine kinase Mirk/dyrk1B is a downstream effector of oncogenic K-ras, the most common mutation in this cancer. Mirk expression is elevated in quiescent pancreatic cancer cells and mediates their prolonged survival through increasing expression of a cohort of antioxidant genes. Mirk is expressed in about 90% of pancreatic cancers and is amplified in a subset. Mirk appears not to be an essential gene for normal cells from embryonic knockout studies in mice and RNA interference studies on cultured cells, but is upregulated in pancreatic tumor cells. These unusual characteristics suggest that Mirk may be a selective target for therapeutic intervention.

## 1. Introduction

Pancreatic ductal adenocarcinoma is the fourth leading cause of cancer deaths in the U.S. This poor outcome is caused by the cryptic nature of the disease, which in most patients is only detected when it is inoperable. Pancreatic cancers are strongly resistant to cytotoxic chemotherapeutic drugs and radiation, and have shown only slight response to the drug gemcitabine alone or together with the EGFR inhibitor erlinotib. Although the vast majority of advanced pancreatic cancers have a mutated K-ras gene, clinical targeting of K-ras with farnesyl transferase inhibitors has been unsuccessful (SWOG 9924 study). These dismal results underscore the need for new therapeutic targets in pancreatic cancers, particularly ones which target the numerous out of cycle cancer cells. Analysis of resected tumors for Ki67 antigen, which is present in all phases of the cell cycle except G0, showed only 28 ± 15% of pancreatic cancer cells were positive for nuclear Ki67 in a study of 33 pancreatic adenocarcinomas [1]. Some of the 70% of pancreatic cancer cells out of cycle may be post-mitotic. However, other out of cycle cells are likely to be in a quiescent, reversible G0 or G0-like state, resistant to drugs which target dividing cells, and able to repopulate a tumor. Stem cells from various cancers have been reported to often be quiescent. A G0 state is not simply a long G1 state, but is maintained by a specific program of gene expression. Factors which allow the prolonged survival of quiescent tumor cells *in vivo* can be of clinical relevance, such as the serine/threonine kinase Mirk.

Mirk/Dyrk1B is a member of the Minibrain/dyrk family of kinases [2,3,4] which mediate survival and differentiation in certain normal tissues: skeletal muscle (Mirk/dyrk1B) [5], neuronal cells (Dyrk1A) [2,6], erythropoietic cells (Dyrk3) [7,8], and sperm (Dyrk4) [9]. While Dyrk1A knockout caused embryonic lethality, knockout of Mirk/dyrk1B caused no evident phenotype in mice [10], even in muscle development, suggesting that Mirk is not an essential gene. Supporting this interpretation, normal diploid fibroblasts exhibited no alteration in survival after 20-fold depletion of Mirk [11]. Thus, Mirk appears not to be an essential gene for normal cells, but is upregulated in pancreatic tumor cells where Mirk mediates survival. These unusual characteristics suggest that Mirk may be a selective target for therapeutic intervention.

## 2. Mirk Expression in Pancreatic Cancers

In an immunohistochemical study of Mirk expression, 25 of 28 (89%) pancreatic adenocarcinomas expressed Mirk protein [12]. In 18 of these cases (64%), Mirk protein was present in at least 20% of the cells. In 39% of the tumors (11 of 28), the intensity of staining was elevated and Mirk was present in at least 20% of the cells. In seven of these cases, low level of Mirk protein was also observed in normal acini, but not in normal ducts. Mirk expression was also found to be elevated in pancreatic cancers compared to normal pancreatic ductal epithelium by serial analysis of gene expression (SAGE) using the National Institutes of Health public data base [12]. Elevated Mirk expression was found in skeletal muscle, the normal cell type in which Mirk is most highly expressed [13], and in each of the pancreatic ductal carcinoma cell lines or resected cancers tested by SAGE analysis. However, Mirk transcripts were not detectable in two short-term cultures of normal pancreatic ductal epithelial cells. Thus, measurement of Mirk mRNA levels and Mirk protein levels has shown that Mirk was expressed in about 90% of pancreatic ductal adenocarcinomas, with much lower expression in normal ductal epithelium.

## 3. Mirk Gene Amplification in Pancreatic Cancers

Genetic alterations including mutations, translocations and amplifications can lead to cancer. Such amplicons are maintained in cancers when the amplified genes provide a selective growth or survival advantage. The 19q13 amplicon was first detected as a double minute, and has since been identified in 10–20% of pancreatic cancers and about 30% of ovarian cancers, as well as cases of follicular lymphoma, mantle cell lymphoma, Burkitt’s lymphoma, and both small-cell and non small-cell lung cancer. Several studies have localized this amplicon in pancreatic cancers to a region around 19q13.1–13.2 (reviewed in [14,15]). Mirk/dyrk1B is localized at 19q13.1 [13]. Akt2 is amplified in some pancreatic cancers near this region. However, the Mirk gene was among 16 genes within the consistently amplified 660 kb subregion of the 19q13 amplicon in pancreatic cancers, while the nearby gene Akt2 was not [16], making it very unlikely that the 19q13 amplicon was selected for because of Akt2. There were no proto-oncogenes or survival proteins within the 16 genes, so the amplicon may be maintained by selection for Mirk. Amplified Mirk genes were detected in the Panc1 and SU86.86 pancreatic cancer cell lines [16]; lines used extensively in the following studies.

## 4. Oncogenic K-ras Proteins Activate Mirk [11]

Mirk was an active kinase in each pancreatic cancer cell line in which it was detected [12], but the mechanism of its activation in this cancer was unknown. Mutant K-ras genes are found in about 90% of pancreatic cancers [17] and have been shown to be the initiating lesion in murine models of PDA [18]. A mutant K-ras to Rac1 to MKK3 signaling cascade was shown to activate Mirk by analyzing individual parts of the signaling cascade. Mirk was first shown to be activated by phosphorylation by its upstream activator, the MAP kinase kinase MKK3 [19,20]. Rac1 is known to be one of the effectors of Ras, and Mirk was activated by a constitutively active mutant Rac1QL through MKK3 in transient transfection experiments and by endogenous Rac1 activated by cadherin ligation in nontransformed MDCK cells [21]. These experiments confirmed a Rac1 to MKK3 to Mirk pathway.

**Figure 1:**
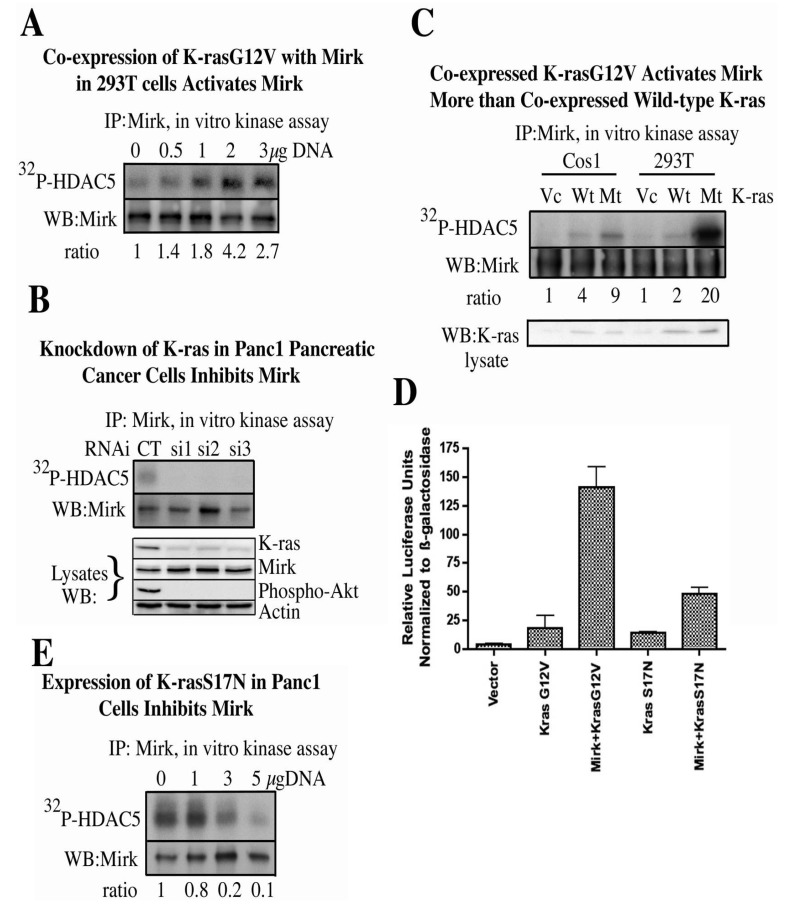
Expression of oncogenic K-ras activates Mirk while depletion of K-ras inhibits Mirk activity. In all studies, as noted, Mirk was immunoprecipitated from the cells using a polyclonal antibody to the unique C-terminus of Mirk. Subsequently, its *in vitro* kinase activity was measured on a specific Mirk substrate, a 1–283 amino acid N-terminal fragment of histone deacetylase 5 (HDAC5) which contains only one Mirk phosphorylation site at serine 279. (**A**). 293T cells were co-transfected with expression plasmids for His-Mirk and increasing concentrations of K-rasG12V. (**B**). K-ras mRNA depleted by three different RNAi duplexes. (**C**). 293T cells and Cos-1 cells were co-transfected with expression plasmids for His-Mirk and either K-rasG12V, wild-type K-ras or vector control. (**D**). The ß-fibrinogen reporter construct (ß-28)_3_-Luc was co-transfected in Cos1 cells with expression plasmids for HNF1, oncogenic K-rasG12V or dominant negative K-rasS17N, and Mirk, as noted. **E.** Increasing amounts of expression plasmid for dominant negative K-rasS17N expressed in Panc1 cells. (Reprinted with permission from [11]).

The ability of one oncogenic mutant K-ras (K-rasG12V) to initiate this signaling pathway and activate Mirk was then assayed (Figure 1A). Mirk was co-expressed with increasing amounts of K-rasG12V expression plasmid. Mirk was then immunoprecipitated and its kinase activity measured in an *in vitro* kinase assay using a known *in vivo* substrate of Mirk, HDAC5 (histone deacetylase) [22]. An *N*-terminal fragment of HDAC5 was utilized that contains one Mirk phosphorylation site at Ser279, with the casein kinase II site of Ser259 mutated to alanine [22]. Expression of increasing amounts of K-rasG12V led to a dose-dependent increase in Mirk kinase activity (Figure 1A) [11]. The relative abilities of mutant K-ras and wild-type K-ras to activate co-expressed Mirk were then compared. K-rasG12V increased Mirk activity a mean of five-fold more than wild-type K-ras in transient transfection experiments (Figure 1C) [11]. 

The results of these direct kinase assays were confirmed by using an HNF1 (hepatocyte nuclear factor 1) reporter assay. Mirk has been shown to increase HNF1 transcriptional activity by two-to-three-fold [19]. HNF1 and the HNF1 reporter were co-transfected with either the active K-rasG12V mutant or a dominant negative K-rasS17N mutant. Both of these K-ras mutants induced the same slight increase in reporter activity over background in the absence of exogenous Mirk (Figure 1D) [11], which demonstrated that the modest increase in HNF1 activity was unrelated to the GTPase, oncogenic functions of K-rasG12V. Co-expressed wild-type Mirk introduced a further eight-fold increase in reporter activity only in the presence of K-rasG12V. The two-to-three-fold increase in HNF1 reporter activity observed in cells expressing both Mirk and dominant negative K-rasS17N was no more than that induced by Mirk alone in earlier studies [19]. Thus, this reporter assay and direct kinase activity assays demonstrated that oncogenic K-ras proteins lie upstream of Mirk in a signaling cascade. 

## 5. Depletion of K-ras or Expression of a Dominant Negative K-ras Inhibits Mirk Activity in Pancreatic Carcinoma Cells [11]

Panc1 cells exhibit an endogenous activating mutation in K-ras and also express activated Mirk [12]. We questioned whether depletion of oncogenic K-ras in these cells would inhibit the activity of endogenous Mirk. K-ras mRNA was depleted by 50–70% in Panc1 cells by transfection of three synthetic duplex RNAi’s to different regions of the K-ras mRNA (Figure 1B, lower panel) [11]. Analysis of the cell lysates also demonstrated a dramatic decrease in activation of one effector of oncogenic K-ras, the survival kinase Akt, as determined by Western blotting for Akt phosphorylated at Ser473. Equivalent amounts of endogenous Mirk were immunoprecipitated from each culture and Mirk kinase activity determined in an *in vitro* kinase assay on a fragment of HDAC5. Depletion of K-ras by each RNAi was enough to decrease Mirk kinase activity to undetectable levels although there was no decrease in Mirk protein levels, as determined by western blotting of total cell lysates (Figure 1B). Therefore, even partial depletion of endogenous K-ras levels in a cell line expressing oncogenic K-ras was enough to dramatically decrease Mirk kinase activity.

Expression of a dominant negative K-ras gene, K-rasS17N, was performed by transient transfection of two pancreatic cancer cell lines with endogenous K-ras mutations, Panc1 (Figure 1E) and SU86.86 [11]. Overexpression of total K-ras proteins in the cells due to transfection was detected by Western blotting [11]. Equivalent amounts of endogenous Mirk were immunoprecipitated from each culture and Mirk kinase activity was determined in an *in vitro* kinase assay on HDAC5. In both cell lines, increased expression of the dominant negative S17N mutant led to a dose-dependent decrease in Mirk activity: to 10–20% of the control levels in Panc1 cells (Figure 1E). Therefore, Mirk activity was strongly inhibited by either depletion of endogenous oncogenic mutant K-ras by each of three different synthetic RNAi’s or by inhibition of endogenous oncogenic mutant K-ras by transient overexpression of a dominant negative construct. These experiments provide evidence that Mirk is a novel downstream effector of endogenous oncogenic K-ras in two pancreatic cancer cell lines.

## 6. Depletion of Either Mirk or K-ras Decreases Anchorage-Dependent Colony Formation in Pancreatic Cancer Cells [11]

**Figure 2:**
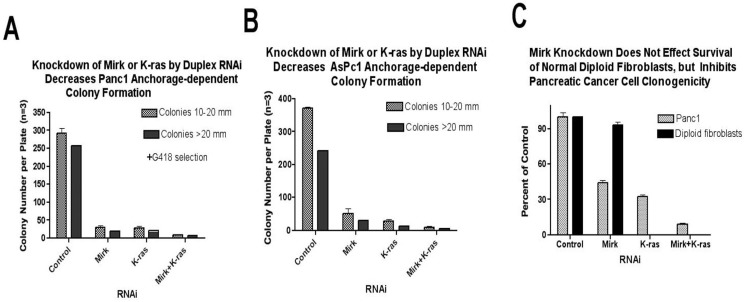
Depletion of either Mirk or K-ras inhibits anchorage-dependent colony formation in pancreatic cancer cells. (**A**). Panc1 cells were depleted of Mirk, K-ras or both Mirk and K-ras by RNA interference, and pcDNA3-neoR was co-transfected to enable selection of the transfected cells. The transfected cells were maintained in serum-free medium for two days, and then allowed to proliferate in growth medium plus G418 for three weeks for colony growth. (**B**). Colony formation as in A with AsPc1 pancreatic cancer cells. (**C**). Experiment as in A with Panc1 cells without co-transfected pcDNA-neoR. Normal diploid BJ fibroblasts were depleted of Mirk, plated at 2000 cells per 100 mm dish, and then allowed to grow into cell clusters for three weeks. Approximately 1500 cell clusters were seen in the control. G418 selection was not employed for either cell type. For all: Mean ± S.D. is shown (n = 3). (Reprinted with permission from [11]).

Colony formation assays test for the most aggressive cells within a tumor cell line. Mirk was depleted from Panc1 pancreatic cancer cells by RNAi with the control a GC-matched RNA duplex. Panc1 cells were maintained in serum-free medium for two days to decrease the activity of serum-factor initiated survival pathways such as IGF-1/Akt or IGF1/STAT3. Mirk levels and Mirk activity increase in serum-free culture because of upregulation of Mirk mRNA [23,24], so this stress condition might be expected to increase reliance on Mirk. Cells were then switched to serum-containing growth media containing G418 for three weeks to allow formation of colonies from the surviving cells. The transfected clonogenic cells also expressed the neomycin resistance gene because the pcDNA3 vector was co-transfected with the synthetic RNAi duplexes at the time of Mirk depletion. Depletion of Mirk in Panc1 cells led to a 10-fold decrease in the number of colonies 10–20 mm in diameter and a 14-fold decrease in the larger colonies (Figure 2A). Depletion of K-ras induced a mean 12-fold decrease in colony formation, while depletion of both K-ras and Mirk induced a much larger 37-fold decrease (Figure 2A). Oncogenic K-ras initiates multiple survival pathways such as PI3-kinase/Akt and MEK/Erk in addition to Mirk, so depletion of K-ras would be expected to have a greater effect than depletion of Mirk alone. Similar studies using transfection of a pSilencer vector targeting other sequences also showed that Mirk depletion decreased clonogenicity of Panc1 cells [12]. Thus, two different methods to reduce Mirk expression by RNA interference, directed to different sequences, led to a large loss in Panc1 cell clonogenicity. As another control, colony formation in Panc1 cells was determined without selection for the transfected cells. Depletion of Mirk or K-ras by synthetic RNAi duplexes without selection for the co-transfected resistance marker led to a two-fold to three-fold loss of colony formation, respectively, while depletion of both genes reduced colony formation 11-fold (Figure 2C). Thus, even under conditions in which nontransfected cells remained in the cultures, a reduction in clonogenicity was seen. 

Mirk levels vary greatly among resected cancers and within pancreatic cancer cell lines. The Panc1 cells and SU86.86 cells with amplification of the Mirk gene express high levels of Mirk protein, while AsPc1 cells express low Mirk levels. However, depletion of Mirk in AsPc1 cells did increase apoptosis of these cells indicating that Mirk has a role in survival of at least a subpopulation of AsPc1 cells [12]. The effect of depletion of Mirk, K-ras or both genes on AsPc1 colony formation was determined, with selection for the transfected cells (Figure 2B). Depletion of Mirk reduced AsPc1 colony formation eight-fold, while depletion of K-ras was more effective, reducing colony formation 17-fold. Depletion of both genes reduced colony formation 50-fold. Thus, Mirk helps to mediate survival of the subpopulation of AsPc1 cells which are clonogenic. 

## 7. Mirk Knockdown in Normal Diploid Fibroblasts [11]

Mirk/dyrk1B knockout mice are viable, which suggests that Mirk is not essential for the survival of nontransformed cells [10]. To test this hypothesis, Mirk was depleted from human normal diploid fibroblasts, and cell growth and survival was then assayed under anchorage dependent conditions at low plating density. Treatment of BJ human diploid fibroblasts with the synthetic RNAi duplexes led to a 25-fold decrease in Mirk protein levels (data not shown). However, this large loss in Mirk levels caused no apparent decrease in the growth of normal diploid fibroblasts (Figure 2C) [12]. The BJ fibroblasts do not form colonies like the tumor cells, but grow into small cell clusters. Thus, depletion of Mirk did not inhibit the survival of diploid fibroblasts plated at low cell density, while inhibiting the clonal growth of pancreatic cancer cells. Possibly, diploid fibroblasts depend for survival under the stress conditions of low plating density on many anti-apoptotic signals, including those initiated by integrins which mediate cell attachment. In contrast, survival of clonogenic pancreatic tumor cells was largely dependent on oncogenic K-ras and Mirk kinase. 

## 8. Mirk Can Be Activated through a K-ras to Rac1 Signaling Pathway [11]

In earlier studies, Mirk had been shown to be activated by overexpressed constitutively active mutant Rac1QL and by endogenous Rac1 activated by cadherin ligation [21]. The GTP-Rac1 forms, which bound to the Rac binding domain of Pak1 coupled to agarose beads, were quantified and compared to total Rac1 in pancreatic cancer cell lysates by Western blot analysis. Rac1 was activated in pancreatic carcinoma cell lines with oncogenic K-ras genes and in BxPc3 cells with wild-type K-ras genes [11].

**Figure 3:**
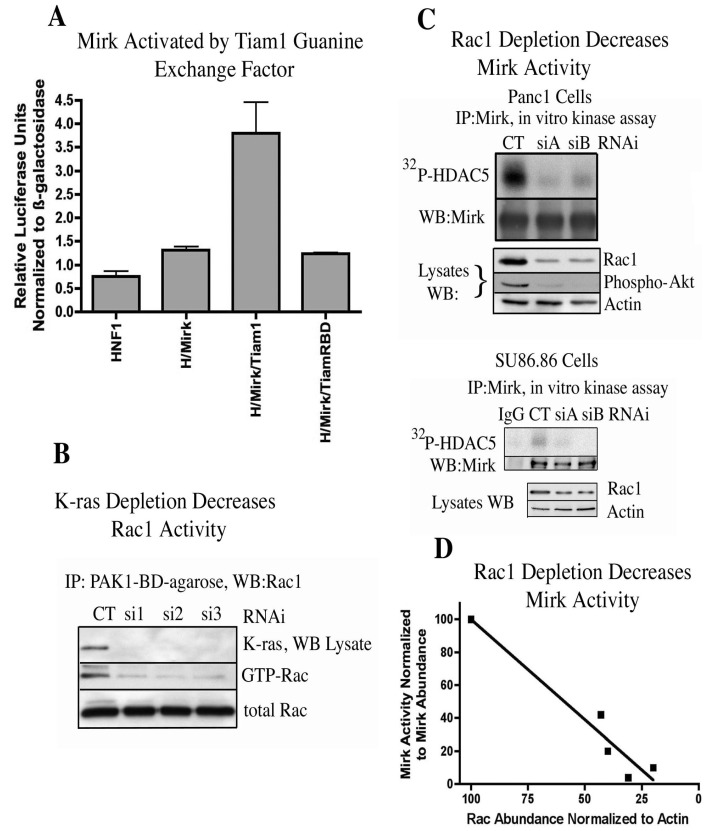
Mirk is activated through K-ras to Rac1 signaling. (**A**). The reporter construct (β-28)_3_-Luc was co-transfected with expression plasmids for HNF1 (H), Mirk and either Tiam1 or Tiam1 mutated at its ras-binding domain (TiamRBD). (**B**). K-ras mRNA was depleted in Panc1 cells, then GTP-Rac1 assayed by GST-pulldown with GST-PAK1-binding domain-conjugated to agarose, followed by Western blotting for Rac1. (**C**). Rac1 mRNA was depleted in Panc1 (upper panel) or SU86.86 (lower panel) cells by two different RNAi duplexes, and Mirk kinase activity determined as in Figure 1. (**D**). Linear regression analysis of data from panel C gave an r squared value of 0.9381. (Reprinted with permission from [11]).

Rac1 itself can be activated by Ras proteins through the Rac-specific guanine nucleotide exchange factor Tiam1 [25]. The capacity of exogenous Tiam1 to activate Mirk in HNF1 reporter assays was determined. Tiam mutated at His798 and its flanking region had been shown to exhibit a much poorer binding affinity to oncogenic Ras proteins than wild-type Tiam 1 [25] and was used as the control. Tiam1 induced a 4-fold increase in Mirk activation in 293T cells (Figure 3A) and a two-fold increase in Mirk activation in Panc1 cells [11] while the Tiam RBD (ras binding domain) mutant induced no activation of Mirk. Since Tiam1 [26] and Rac1 are active in the majority of pancreatic cancer cells, Tiam1 might mediate the activation of Rac1. The hypothesis was next tested that oncogenic K-ras found in Panc1 cells was responsible for the activation of Rac1 seen in these cells. Three synthetic duplex RNAi’s to different regions of the K-ras mRNA were next used to determine whether depletion of K-ras would extinguish Rac1 activation. Each of the three RNAi’s depleted K-ras a mean of 14-fold in Panc1 cells (Figure 3B). In each case, this substantial depletion of K-ras was followed by a mean five-fold decrease in the activation of Rac1. Therefore, oncogenic K-ras activated Rac1 in Panc1 cells, and was probably responsible, at least in part, for the activation of Rac1 seen in the other pancreatic cancer cell lines with mutant K-ras genes.

## 9. Mirk Activity in Panc1 Pancreatic Cancer Cells Is Inhibited by Either Depletion of Rac1 by RNA Interference or Pharmacological Inhibition of Rac1 [11]

Rac1 mRNA was depleted in Panc1 pancreatic cancer cells and in SU86.86 pancreatic cancer cells by two synthetic duplex RNAi’s to different regions of the Rac1 mRNA (Figure 3C). Analysis of total cell lysates by Western blotting demonstrated that siA and siB depleted Rac1 protein levels. Depletion of Rac1 in Panc1 cells led to a marked decrease in activity of a downstream effector, Akt, as shown by a decrease in Akt phosphorylation. Mirk was then immunoprecipitated from the Rac1 depleted cells and its kinase activity measured by an *in vitro* kinase reaction on HDAC5. Depletion of Rac1 levels by siA and siB led to a strong decrease in Mirk activity in both cell lines (Figure 3C, upper lanes of both panels). Linear regression analysis was employed to demonstrate the relationship between depletion of Rac1 and inhibition of Mirk activity (Figure 3D) by pooling the data from depletion of Rac1 in both Panc1 and SU86.86 cells. Depletion of Rac1 was linearly related to a decrease in Mirk activity. The cell permeable Rac1 inhibitor NSC23766 was used in a time-course study to compare inhibition of Rac1 with inhibition of Mirk kinase activity, with the data showing a sigmoidal dose-response relationship by nonlinear regression analysis [11]. Therefore, both depletion of Rac1 by RNA interference and pharmacological inhibition of Rac1 inhibited Mirk kinase activity in pancreatic cancer cells. These experiments (Figure 1, Figure 3), taken together, show that oncogenic K-ras activates Mirk as a kinase through a Rac1 to MKK3 to Mirk signaling cascade, and that Mirk mediates some of the survival functions of oncogenic K-ras for clonogenic growth (Figure 2).

## 10. Quiescent Pancreatic Cancer Cells, Both Cell Lines and Resected Cancers, Exhibit Elevated Levels of Mirk

**Figure 4:**
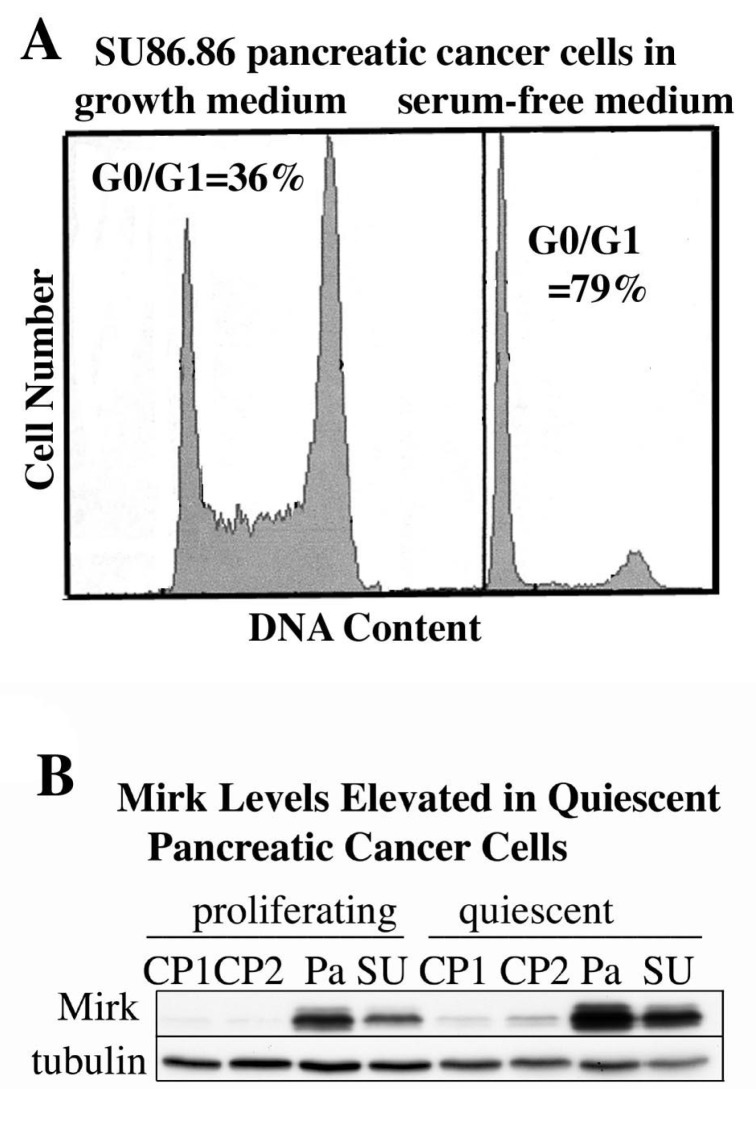
Mirk levels are elevated in quiescent pancreatic cancer cells. (**A**). SU86.86 cells were cultured for 48 h in medium containing either 7% fetal calf serum to maintain proliferation, or no serum mitogens to induce quiescence, then analyzed for cell cycle position by flow cytometry after staining with propidium iodide. (**B**). Panc1 (Pa), SU86.86 (SU), Capan1 (CP1), and Capan 2 (CP2) were cultured as in A before Western blotting.

Culture of some tumor cells in serum-free medium has been reported to place them in quiescent, non-cycling state. Proliferating SU86.86 pancreatic cancer cultures contained 36% of cells in G0/G1, while culture in serum-free medium to induce quiescence increased the fraction of cells in G0/G1 to 79% (Figure 4A). Culture of each of four pancreatic cancer cell lines in serum-free medium to induce quiescence led to an average three-fold increase in Mirk protein levels (Figure 4B). Mirk protein was rapidly lost when cells entered the cycle in response to serum mitogens because of transcriptional downregulation through the MEK/Erk pathway in both cancer cells and nontransformed cells [23].

The increase in Mirk expression in quiescent pancreatic cancer cells was not limited to cell lines. Mirk protein had been detected by immunohistochemistry in 25 of 28 resected cases of pancreatic ductal adenocarcinoma, with elevated expression in 11 cases [12]. Three of these cases were recut and sequential sections analyzed for Mirk expression and for Ki67 [27], a nuclear protein reported to be absent in G0 cells, but present in cycling cells in G1, S and G2. Tumor cells were not uniformly positive for Mirk, but ranged from a few percent to 20–70% positive. The same malignant ducts on each slide could be readily ascertained because of their similarities in size and general morphology. However, individual cells in these ducts could not be reliably identified on the adjacent slides so all of the cells in the same malignant ducts were counted for Mirk reactivity and for Ki67 nuclear staining. In two cases, almost all of the malignant ductal epithelium was positive for Mirk and negative for Ki67 nuclear staining (0 of 352), (0/140). Thus these non-cycling tumor cells expressed Mirk. In another case, there were a group of closely associated malignant ducts which exhibited elevated expression of Mirk and no Ki67 staining (0/67 cells) while two proliferating lymphocytes directly adjacent exhibited nuclear Ki67 staining. Thus, in each of three cases [11], pancreatic cancer cells which expressed Mirk protein detectable by immunohistochemistry were out of cycle, and possibly quiescent. 

## 11. When Pancreatic Cancer Cells Accumulate in the G0 Quiescent State, Mirk Levels Are Increased Several Fold [27]

**Figure 5:**
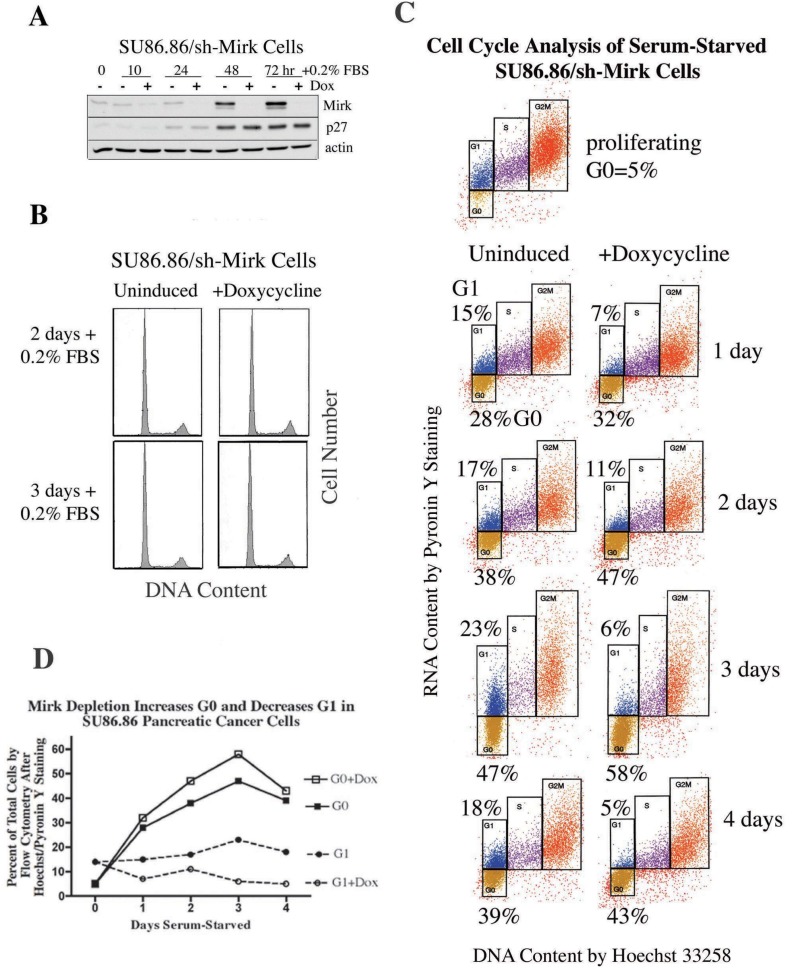
Serum-starved SU86.86 pancreatic cancer cells become quiescent in G0, and exhibit cell cycle modulations when depleted of Mirk. (**A**). Lentiviral stably transfected SU86.86/sh-Mirk pancreatic cancer cells expressing an inducible shRNA that targets the Mirk mRNA were cultured with doxycycline to induce the construct before Western blotting for Mirk, p27kip1 and actin. (**B**). Parallel cultures to panel A were analyzed by flow cytometry with propidium iodide staining. (**C**). Cell cycle position was determined by cytofluorography on ethanol-fixed SU86.86/sh-Mirk cells sequentially stained with Hoechst 33258 for DNA (x-axis) and then Pyronin Y (y-axis) for RNA. Cells were either proliferating in growth medium or serum-starved, shMirk induced by doxycycline as noted. G0 (gold, lower boxes) and G1 (blue, upper boxes) cells exhibit 2N DNA, with S phase cells (purple) and G2+M cells (orange). The flow cytometry data was performed on the same day under the same gating conditions. (**D**). Alterations in the fraction of cells in G0 and G1 with Mirk depletion. (Reprinted with permission from [27]).

Quiescent tumor cells in G0 are considerably less responsive than cycling cells to chemotherapeutic drugs and radiation and may be one source of recurrent tumors, therefore, the role of Mirk in SU86.86 pancreatic cancer cells in G0 was determined. The SU86.86 line was chosen because it contains an amplified Mirk gene within the 19q13 amplicon [16], and depletion of Mirk in these cells decreased anchorage-independent colony formation and enhanced apoptosis by the chemotherapeutic drug gemcitabine [12]. SU86.86 cells stably bearing a doxycycline-inducible shRNA to the Mirk mRNA (SU86.86/sh-Mirk) were utilized to assess the role of Mirk in G0 cells. Mirk protein levels increased seven-fold in the uninduced cells after culturing for three days in DMEM supplemented with only 0.2% FBS to inhibit cell cycling (Figure 5A). Parallel analysis of cell cycle position by flow cytometry with propidium iodide staining demonstrated that after 2–3 days about 70% of SU86.86/sh-Mirk cells with high Mirk levels had accumulated predominantly with a 2N DNA content, either in G0 or G1. Induction of the short hairpin RNA directed to the Mirk mRNA prevented any increase in Mirk levels, which were 25-fold lower compared with the uninduced cells, but only slightly decreased the fraction of cells in G0/G1 as assessed by propidium iodide staining for DNA content (mean of 71% to 67%, Figure 5B). Thus. Mirk is not essential for pancreatic tumor cells to accumulate in a quiescent state. The levels of the CDK inhibitor p27kip1, which helps to maintain a G0 arrest, were increased several-fold during culture in low serum medium and this increase was not altered by Mirk depletion (Figure 5A).

Quiescent cells in G0 are characterized by lower levels of RNA than that seen in G1 and S phase cells and were detected by two-parameter analysis by flow cytometry using sequential staining with Hoechst dye to bind DNA and then Pyronin Y to bind to RNA. Proliferating cultures contained 5% of cells in G0, but after one day of serum-starvation, 28% of the pancreatic cancer cells were in G0 and this fraction increased to a mean of 38% (±4%) between days 2 and 4 (Figure 5C). When Mirk was depleted, the fraction of cells in G0 between days 2 and 4 increased further to a mean of 47% (±4%). An average 22% increase in the fraction of cells in G0 was seen over the five time points (Figure 5D). Thus, quiescent SU86.86 pancreatic cancer cells could enter G0 when Mirk was depleted, and more cells did, suggesting that Mirk had functions in G0. 

## 12. Pancreatic Cancer Cells Maintained in G0 Quiescence without Mirk Lose Viability [27]

**Figure 6:**
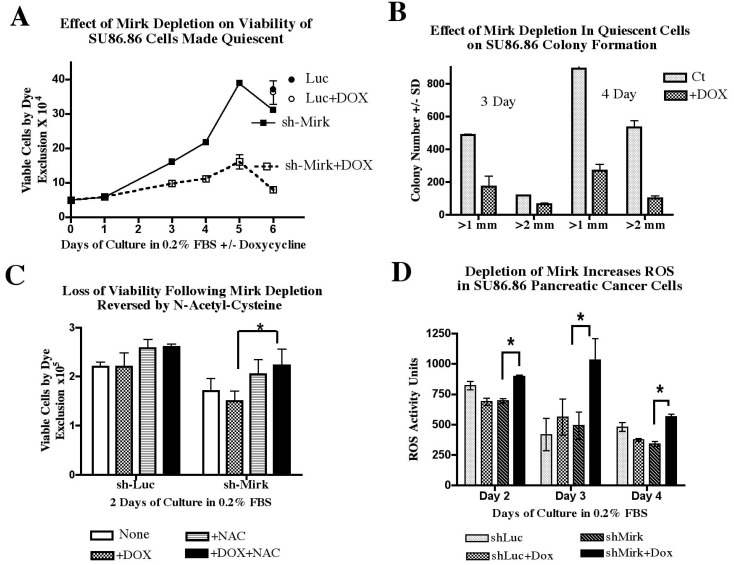
Mirk depletion leads to loss of viability and an increase in ROS in quiescent SU86.86 cells. (**A**). Viability by trypan blue exclusion assay: SU86.86/sh-Mirk and control SU86.86/luc (luciferase gene) cells were cultured for up to six days in medium containing 0.2% FBS to induce a quiescent state. Viable cells were counted using a phase microscope after staining with trypan blue dye. Mean ± SEM shown if SEM > 5%. (**B**). Viability by colony formation assay: Colonies arising from SU86.86/sh-Mirk cells made quiescent 3 or 4 days, then grown in DMEM containing 10% FBS without doxycycline, were counted with an automatic colony counter, selecting for colonies of diameters of at least 1 mm or 2 mm. (**C**). SU86.86/sh-Mirk and control SU86.86/luc cells were made quiescent for two days and then assayed for viable cells, as in panel A, with the addition of the antioxidant N-acetyl-cysteine (NAC) at 1 mM as noted. (**D**). SU86.86/sh-Mirk cells and control SU86.86/Luc cells were made quiescent and their ROS species detected with CM-H_2_DCFA using a fluorometer. (Reprinted with permission from [27]).

What was the role of Mirk in quiescent tumor cells in G0? When Mirk was depleted in SU86.86/sh-Mirk cultures made quiescent and enriched in G0 cells (Figure 5C) by serum-starvation, cells lost viability as shown by their decreased capacity for trypan blue exclusion (Figure 6A) and their decreased ability to sustain clonogenic growth (Figure 6B). Cultures of SU86.86/sh-Mirk cells depleted of Mirk by the inducible shRNA contained about half as many viable cells after three days in low serum medium (Figure 6A) when 47ߝ58% of cells were accumulated in G0 (Figure 5C). With continued culturing, the control, uninduced cells cycled slowly as the total number of viable cells more than doubled from three to five days of culture. In contrast, little increase in viable cell number was seen in the Mirk-depleted cultures. After six days, four-times as many viable cells were found in the uninduced cultures compared with the Mirk-depleted ones (Figure 6A). The majority of induced and uninduced cells accumulated in G0 or G1 during serum starvation up to seven days, but cells were continually dying during this period. The number of nonviable, trypan blue positive cells counted on day 4 constituted 23% of the total uninduced cells, but constituted a greater percentage, 41% of total cells, in the Mirk-depleted cultures, when about 40% of both cultures were in G0 (Figure 5C). Thus, more cell death occurred when cells were maintained in G0 quiescence in the absence of Mirk. The control cells for this study were SU86.86/Luc cells stably expressing an inducible short hairpin to the unexpressed gene luciferase. SU86.86/Luc cells were cultured with or without 1 µg/ml doxycycline for six days and their viability assayed by trypan blue exclusion. There was no difference in the number of viable cells between the control and doxycycline-treated SU86.86/Luc cultures (Figure 6A), demonstrating that the loss in cell viability by doxycycline-induced depletion of Mirk was due to loss of Mirk expression, not due to toxicity by doxycycline. 

The viability of the Mirk-depleted quiescent cultures was also determined by assaying their capacity for colony formation. SU86.86/sh-Mirk cells were cultured for three or four days in medium supplemented with 0.2% FBS to place the majority of control cells and Mirk-depleted cells in either G0 or G1. The cells were then trypsinized, equally diluted and replated in serum-containing growth medium in the absence of doxycycline. Colonies which arose 10 days after plating were counted and displayed by colony size (Figure 6B). Quiescent cells depleted of Mirk gave rise to an average of 33% (±4%) as many colonies as controls. Thus, depletion of Mirk in cultures of quiescent pancreatic cancer cells reduced their viability three- to four-fold by two different assays, direct measurement of viable cells capable of dye exclusion and measurement of their capacity to maintain clonogenic growth.

## 13. Mirk Mediates Pancreatic Cancer Cell Survival by Decreasing the Level of Reactive Oxygen Species (ROS) [27]

To test the hypothesis that Mirk controlled ROS levels in pancreatic cancers, SU86.86/sh-Mirk cells and control SU86.86/sh-Luc cells were cultured in DMEM supplemented with 0.2% FBS for two, three and four days to accumulate cells in G0 and G1. Depletion of Mirk strongly correlated with increased ROS activity over this period by a statistically significant amount (p = 0.0488 by student’s paired t test), with increases after two, three or four days of 129%, 210% and 166%, respectively (Figure 6D). The increase in ROS activity was less after four days when cell death was evident in the cultures (Figure 6A). Possibly cells with the highest ROS levels had died at this time point. In contrast, no increase in basal ROS activity was seen at any time point with doxycycline treatment of the control SU86.86/Luc cells (p = 0.3776). A similar increase in ROS was seen when the parental SU86.86 cells were depleted of Mirk by transient transfection of RNAi duplexes (data not shown). 

Elevated ROS levels were shown to be one cause of the loss of viability seen when Mirk was depleted. SU86.86 cells were incubated with the antioxidant *N*-acetyl cysteine (NAC) and their viability and their ROS activity were compared with and without depletion of Mirk. Incubation with NAC for 48 hours decreased by about half the ROS levels in both the uninduced and in the doxycycline-induced SU86.86/sh-Mirk cells (data not shown), with both reductions statistically significant with p < 0.001. Addition of NAC to Mirk-depleted quiescent cells led to a 50% increase in viable cell numbers as assayed by dye exclusion (Figure 6C), and partially restored clonogenicity to the Mirk-depleted cells a statistically significant effect with p < 0.001 [27]. To assess the generality of these results, Mirk was depleted from another pancreatic cancer cell line, Panc1, by transient transfection of RNAi duplexes directed to a different sequence in the Mirk mRNA than that targeted by the shRNA. Both the shRNA and the synthetic RNAi duplexes greatly reduced Mirk mRNA levels. Parallel time-courses showed that 10-fold Mirk depletion was seen 48 to 72 hours post-transfection, while ROS levels increased at the same time in only the Mirk-depleted cultures [27]. Depletion of Mirk by two different methods in two pancreatic cancer cell lines led to an increase in ROS. Thus, one role of Mirk is to maintain the viability of quiescent tumor cells by decreasing ROS levels. 

## 14. Mirk Mediates Cell Survival by Increasing Transcription of Genes Which Reduce ROS Levels [27]

Since Mirk is a co-activator of several transcription factors (HNF1alpha, MEF2C, FOXO3a, FOXO1a) by various mechanisms [19,22,28,29], possibly Mirk enhanced the survival of pancreatic cancer cells by upregulating expression of genes involved in countering oxidative damage. A microarray screen compared gene expression profiles in Mirk-depleted (via doxycycline-inducible shRNA) SU86.86 pancreatic cancer cells *versus* uninduced controls. No decrease in expression of anti-apoptotic or DNA repair genes was seen, but decreased expression of several antioxidant genes followed Mirk depletion. 

**Figure 7:**
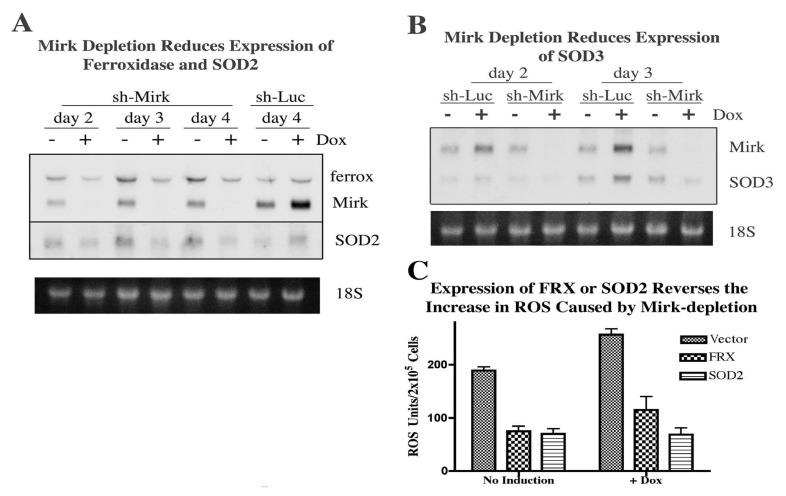
Mirk increases the transcription of antioxidant genes. **A** and **B**. SU86.86/sh-Mirk cells and control SU86.86/Luc cells were made quiescent for two to four days. Northern blotting was performed for levels of Mirk, ferroxidase, SOD2, and SOD3 mRNA. Ethidium bromide stained gels are shown below. **C**. SOD2 and ferroxidase genes were transfected into quiescent SU86.86/sh-Mirk with doxycycline added as noted for two days before measurement of ROS levels. (Reprinted with permission from [27]).

The microarray data were confirmed by northern analysis of mRNA levels of ferroxidase, superoxide dismutase 2 (SOD2), and SOD3 following culture in DMEM supplemented with 0.2% FBS for two to four days. Mirk levels were decreased over 10-fold by doxycycline-induction of shRNA over this induction period (Figure 7A,B), leading to a 2.5 fold (±0.8) mean reduction in ferroxidase mRNA levels and a 3 fold (±0.2) mean reduction in SOD2 and SOD3 mRNA levels. Induction of the shRNA to the luciferase gene was used as a control, and surprisingly led to a two-fold increase in Mirk mRNA levels on days 2, 3 and 4, which correlated with small increases in ferroxidase, SOD2, and SOD3 mRNA levels on these days (Figure 7A,B, data not shown). Thus, when mRNA levels of Mirk were reduced, mRNA levels of ferroxidase, SOD2 and SOD3 were also reduced, while small increases in Mirk expression correlated with small increases in expression of these antioxidant genes. In the control cells ferroxidase protein levels were up to six–fold higher over Mirk-depleted cells (mean 3.3 fold ± 0.8, n = 4). Likewise, levels of SOD2 and SOD3 were on average 1.5 fold (±0.1, n = 6) higher in uninduced cells compared with Mirk-depleted cells after three–five days of culture in low serum medium. As a control, ferroxidase, SOD2, and SOD3 levels were compared in SU86.86/luc cells. No decreases in levels of these proteins were seen after 3–5 days treatment with doxycycline. These three genes function together to maintain low levels of superoxide and hydroxyl ions. SOD2 and SOD3 detoxify superoxide ions to hydrogen peroxide which is then metabolized to water when its conversion to free hydroxyl ion is blocked by ferroxidase (see Section 15: Significance).

To confirm that reduction of expression of the antioxidant genes SOD2 and ferroxidase were able to cause the increase in ROS levels seen when Mirk was depleted, the SOD2 and ferroxidase genes were transiently transfected into SU86.86/shMirk cells. Depletion of Mirk for two days caused a 36% increase in ROS levels, similar to that seen in earlier experiments (compare Figure 7C and Figure 6D). However, expression of either SOD2 or ferroxidase reduced ROS levels over two-fold in both Mirk-depleted cells and in untreated cells (Figure 7C). Therefore, Mirk upregulates the transcription of antioxidant genes which maintain the viability of pancreatic cancer cells in G0 by reducing ROS levels.

## 15. Significance

Mutant K-ras has been implicated in mediating survival in several different types of cancer cells. The kinase Mirk/dyrk1B is activated by oncogenic K-ras in pancreatic cancer cells and mediates their survival [11], at least in part, through lowering ROS levels by increasing transcription of at least three antioxidant genes. Culture of epithelial cells under serum-free conditions can increase their intracellular ROS concentrations [30,31], while oncogenic ras proteins increase basal ROS levels as well [32]. SU86.86 and Panc1 pancreatic cancer cells exhibit amplified Mirk genes, and were mutant in their K-ras genes, so would be expected to exhibit elevated ROS levels. Mirk protein levels were found to increase seven-fold in quiescent cultures where the cells had accumulated in G0, and depletion of Mirk in quiescent cells decreased their viability four-fold. G0 tumor cells were identified by 2N DNA content and lower abundance of RNA than G1 cells and S phase cells by two-parameter flow cytometry. Thus, Mirk is an unusual kinase that is most active in resting, quiescent cancer cells and mediates the survival of such cells. Identification of potentially druggable targets in quiescent tumor cells is important because such cells may be one source of recurrent tumors. Quiescent cells have been reported to be relatively resistant to apoptosis induced by DNA damage, nutrient starvation or oxidative stress. Normal diploid fibroblasts carrying the bacterial lacI gene in a lambda shuttle vector have been used to quantitate DNA damage and repair in quiescent and in proliferating cells in primary culture. *N*-ethyl-*N*-nitrosourea treatment of such quiescent normal diploid fibroblasts led to less pre-mutagenic damage in the transgene and one-fifth as many mutations, compared with proliferating cells. Quiescent cells were proficient in transcription-coupled repair [33,34], demonstrating their enhanced survival advantage.

G0 cells comprised about 5–10% of the cells in proliferating pancreatic cancer cell cultures in multiple experiments. Tumor cells can enter a final G0 arrest like myeloid leukemia cells undergoing terminal differentiation [35]. Alternatively, tumor cells may be able to enter a G0 period to repair damage occurring from ROS generated by metabolic activity, and then resume cycling. The 10-fold decrease in colony forming ability exhibited by Mirk-depleted SU86.86 cells (Figure 2) suggested that a large fraction of the clonogenic subpopulation of tumor cells were unable to repair ROS-initiated damage in the absence of Mirk in a transient G0 repair period. ROS are oxygen-containing chemical species with reactive chemical properties, such as hydroxyl radicals which contain an unpaired electron and the free radical superoxide. Cancer cells often exhibit higher levels of ROS than normal cells because of increased metabolism and oncogenic stimulation, so are under increased oxidative stress. Genes which detoxify superoxide (superoxide dismutases 2 and 3) and which prevent the generation of hydroxyl radical (ferroxidase/ceruloplasmin) were upregulated in SU86.86 pancreatic cancer cells through Mirk. These genes work together to reduce ROS. 

## 16. Summary

Quiescence of hematopoietic stem cells is maintained by repressing the production of reactive oxygen species (ROS) [36], and thus lowering oxidative stress damage. Our group has recently shown that the serine/threonine kinase Mirk increases transcription of a cohort of genes which detoxify the ROS superoxide and which prevent the generation of hydroxyl radicals [27]. This antioxidant function may provide a selective pressure to maintain the upregulation of Mirk seen in about 90% of pancreatic cancers [12], and the amplification of the Mirk gene in a subset of these cancers. 

Cancer cells often are under increased oxidative stress. Mirk upregulates expression of superoxide dismutases 2 and 3 which detoxify superoxide and the ferroxidase ceruloplasmin which prevents the generation of the hydroxyl radical through the Fe^2+^ dependent Fenton reaction [27]. These three genes work together to reduce ROS. If the kinase Mirk was inactivated so it could not lower endogenous ROS levels, further elevation of ROS levels by chemotherapeutic drugs might be sufficient to induce apoptosis in pancreatic cells.

**Figure 8:**
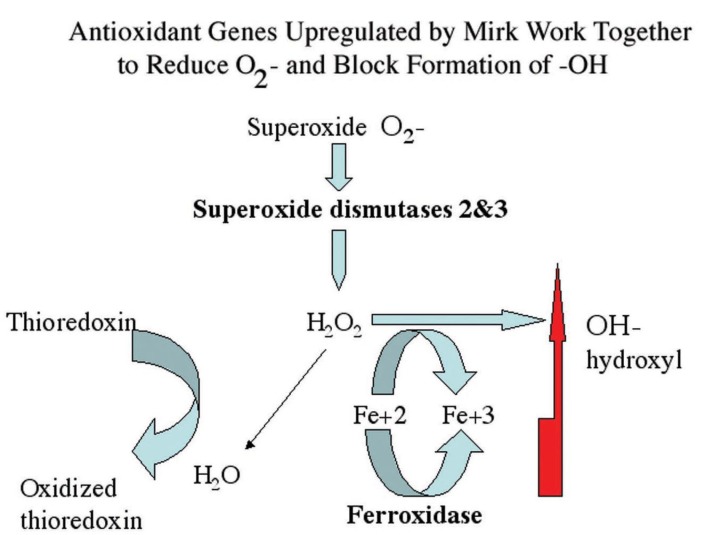
Superoxide dismutases detoxify superoxide resulting in hydrogen peroxide, which in turn can either be metabolized to water or to hydroxyl radical through the Fenton reaction if Fe^2+^ is available. Conversion to hydroxyl radical is blocked by ferroxidase, which converts Fe^2+^ to Fe^3+^. The Mirk-upregulated genes ferroxidase, SOD2 and SOD3 working together increase antioxidant potential while minimizing hydroxyl production.
